# Editorial: Serafino Zappacosta and the Ceppellini School: A Pioneer Model for Nurturing Education in Immunology

**DOI:** 10.3389/fimmu.2020.01524

**Published:** 2020-08-07

**Authors:** Francesca Di Rosa

**Affiliations:** Institute of Molecular Biology and Pathology, Consiglio Nazionale delle Ricerche (IBPM-CNR), Rome, Italy

**Keywords:** education, innate immunity, adaptive immunity, MHC, vaccination

This *Frontiers in Immunology Research Topic* is a collection of articles on the activities and the scientific interests of the founders, faculty, and students of the “Scuola Superiore di Immunologia Ruggero Ceppellini” (Ruggero Ceppellini Advanced School of Immunology), an International School of Immunology founded almost 30 years ago following a pioneer idea by Serafino Zappacosta. The school has more recently become known as the EFIS-EJI Ruggero Ceppellini Advanced School of Immunology founded by Serafino Zappacosta. The re-naming of the school followed the sudden death of Zappacosta in 2006 (1). Furthermore, in 2011 the European Federation of Immunological Societies (EFIS) declared the Ceppellini School one of its regularly sponsored activities. Since then, the European Journal of Immunology (EJI, i.e., the EFIS official journal) has regularly reported on the Ceppellini School's international courses in its “News & EFIS” section [for recent examples see (2–4)].

The opening article of this *Research Topic* is a contribution by Antonio Di Giacomo (Colli Monaldi Hospital, Naples, Italy) who, in 1991, joined Zappacosta (at the time a full professor of immunology at the Federico II University, Naples) in the foundation of the Ceppellini School in Naples, Italy (Di Giacomo). Co-founders were Melchiorre Brai (University of Palermo, Italy), Giovanni B. Ferrara (Federico II University, Naples), Ciro Manzo (Istituto Pascale, Naples), and Alfred Nisonoff (Brandeis University, in Waltham, Massachusetts, USA). The title of Di Giacomo's article, “The Ruggero Ceppellini Advanced School of Immunology and the Neapolitan Scientific Renaissance,” clearly indicates the strong roots of the Ceppellini School in the city of Naples. Di Giacomo illustrated the pioneer vision of the founders and their strong commitment to the generous educational project of the Ceppellini School, which was summarized in the Latin motto suggested by Zappacosta “*non multa sed multum*” (not many but much, i.e., quality not quantity) (Di Giacomo).

The second article is a tribute to Zappacosta by a group of previous students and collaborators who worked with him in Naples, all of them now well-established immunologists in Italy, Europe, and the USA (Carbone et al.). They reported on the research performed by Zappacosta and his team over more than 30 years on the role of MHC in innate and adaptive immunity, showing how their findings contributed to, and often anticipated, key issues of current literature. Silvia Fontana, one of the authors of this perspective, became the President of the Ceppellini School after Zappacosta's death, and her strong commitment and passionate work have been essential for the continuation of the School's educational project. The first author is Ennio Carbone, co-editor of this Research Topic, who sadly died all of a sudden in March 2020 just after he became President of the Ceppellini School. We co-edited this *Research Topic*, but, unfortunately, he could not co-author this editorial. This *Research Topic* is dedicated to his memory.

Zappacosta and colleagues entitled their Advanced Immunology School to Ruggero Ceppellini, an outstanding Italian scientist who gave seminal contributions to the genetics of HLA. Here, Walter Bodmer (University of Oxford, UK) drew a picture of Ruggero Ceppellini and reported about some of his achievements and fruitful insights that inspired his contemporary colleagues and those who followed his path in later years (Bodmer). The inaugural course of the Ceppellini School was on bone marrow transplantation (BMT) in 1992. It was directed by Elizabeth Simpson, at the time working at the Division of Transplantation Biology, MRC Clinical Research Centre, Harrow, Middlesex, UK. In their article for this *Research Topic*, Elizabeth Simpson and Francesco Dazzi (King's College, London, UK) placed the achievements of about six decades of research and clinical experience on BMT in the context of today challenges and discussed how the contributions to the 1992 Ceppellini School course created a remarkable marker point about mid-way between the first BMT in 1957 and current times (Simpson and Dazzi).

In 2006, Stefan Kaufmann, who was Director of the Max-Planck Institute for Infection Biology, Department of Immunology, Berlin, Germany, became Scientific Director of the Ceppellini School. Kaufmann organized and directed several Ceppellini School courses, mostly focused on immune response and vaccination against tuberculosis and other threatening infectious diseases, such as malaria and AIDS (5). Here, Kaufmann gave a historical overview of the most remarkable milestones in immunology, focused on the Nobel laureates' achievements (Kaufmann). This personal and passionate perspective concisely summarized an overwhelming body of work. We also published a commentary by Heniz Kohler (University of Kentucky, Lexington, KY, USA) and colleagues, who integrated Kaufmann's review by emphasizing some additional aspects, for example, the theorical contribution of the idiotypic network theory by Jerne, the thoughtful work on positive and negative selection (of both T and B cells), and the current successes of therapeutic antibodies (Kohler et al.).

Rino Rappuoli, a distinguished vaccinologist who has been part of the faculty of many Ceppellini School courses over the years (5), is co-author, alongside Emanuele Andreano, Ugo D'Oro, and Oretta Finco, of a mini-review discussing the most promising approaches to vaccinology, going from the genome-based “reverse vaccinology” at the end of last century to the “reverse vaccinology 2.0” in 2016 and beyond (Andreano et al.). Siamon Gordon (University of Oxford, UK), Stefan Kaufmann, and Fernando Martinez-Estrada (University of Surrey, UK) were the scientific directors of a memorable Ceppellini School course on tissue phagocytes and function held in 2016 at the Stazione Zoologica “Anton Dohrn,” a research center in Naples where the Russian scientist Elie Metchnikoff (1845–1916), who first described phagocytosis, worked for a short while (2). The contribution by Siamon Gordon and Annette Plüddemann (University of Oxford, UK) to this *Research Topic* is an inspiring discussion on macrophages diversity and function that highlights key open questions on macrophage heterogeneity and provides insights on its underlying pattern (Gordon and Plüddemann).

The last two articles focus on the fruitful sharing of knowledge between young attendees and senior faculty members of some exemplary Ceppellini School courses (4, 6). One article is by Francesco Colucci (University of Cambridge, UK), a Ceppellini School faculty member who was scientific co-director of the 2014 course on the maternal immune system in pregnancy (Colucci). The other article is by three of the participants to the 2018 Ceppellini School course on T-cell memory, i.e., Silvia Piconese (Sapienza University, Rome, Italy), Silvia Campello (University of Rome Tor Vergata, Rome, Italy), and Ambra Natalini (Sapienza University, and Institute of Molecular Biology and Pathology, CNR, Rome, Italy) (Piconese et al.). Both articles give a flavor of the exceptional learning experiences of participants to the Ceppellini School activities.

In 1991, the foundation of the Ceppellini School was a real breakthrough. After almost 30 years, the Ceppellini School continues to be an attractive pole for hundreds of young and enthusiastic participants from Europe, North and South America, the Middle East, Africa, and India. This *Research Topic* aims to offer some historical background and insightful perspectives on the Ceppellini School. Born from a Zappacosta's utopian idea, the school remains dedicated to strongly engaging new generation of young minds.

## Author Contributions

The author confirms being the sole contributor of this work and has approved it for publication.

## Conflict of Interest

The author declares that the research was conducted in the absence of any commercial or financial relationships that could be construed as a potential conflict of interest.
