# Erratum: Editorial: Serafino Zappacosta and the Ceppellini School: A Pioneer Model for Nurturing Education in Immunology

**DOI:** 10.3389/fimmu.2020.600832

**Published:** 2020-09-08

**Authors:** Frontiers Production Office

**Affiliations:** Frontiers Media SA, Lausanne, Switzerland

**Keywords:** education, innate immunity, adaptive immunity, MHC, vaccination

Due to a production error, the citation for “Piconese et al.” was linked to the wrong article. The publisher apologizes for this mistake. The original article has been updated.

